# Bone growth during rapamycin therapy in young rats

**DOI:** 10.1186/1471-2431-9-3

**Published:** 2009-01-13

**Authors:** Cheryl P Sanchez, Yu-Zhu He

**Affiliations:** 1 Department of Pediatrics, University of Wisconsin School of Medicine & Public Health, Madison, Wisconsin, USA

## Abstract

**Background:**

Rapamycin is an effective immunosuppressant widely used to maintain the renal allograft in pediatric patients. Linear growth may be adversely affected in young children since rapamycin has potent anti-proliferative and anti-angiogenic properties.

**Methods:**

Weanling three week old rats were given rapamycin at 2.5 mg/kg daily by gavage for 2 or 4 weeks and compared to a Control group given equivalent amount of saline. Morphometric measurements and biochemical determinations for serum calcium, phosphate, iPTH, urea nitrogen, creatinine and insulin-growth factor I (IGF-I) were obtained. Histomorphometric analysis of the growth plate cartilage, in-situ hybridization experiments and immunohistochemical studies for various proteins were performed to evaluate for chondrocyte proliferation, chondrocyte differentiation and chondro/osteoclastic resorption.

**Results:**

At the end of the 2 weeks, body and tibia length measurements were shorter after rapamycin therapy associated with an enlargement of the hypertrophic zone in the growth plate cartilage. There was a decrease in chondrocyte proliferation assessed by *histone-4 *and *mammalian target of rapamycin *(*mTOR*) expression. A reduction in *parathyroid hormone/parathyroid hormone related peptide (PTH/PTHrP) *and an increase in *Indian hedgehog *(*Ihh*) expression may explain in part, the increase number of hypertrophic chondrocytes. The number of TRAP positive multinucleated chondro/osteoclasts declined in the chondro-osseous junction with a decrease in the *receptor activator of nuclear factor kappa β ligand *(*RANKL*) and *vascular endothelial growth factor *(*VEGF*) expression. Although body and tibial length remained short after 4 weeks of rapamycin, changes in the expression of chondrocyte proliferation, chondrocyte differentiation and chondro/osteoclastic resorption which were significant after 2 weeks of rapamycin improved at the end of 4 weeks.

**Conclusion:**

When given to young rats, 2 weeks of rapamycin significantly decreased endochondral bone growth. No catch-up growth was demonstrated at the end of 4 weeks, although markers of chondrocyte proliferation and differentiation improved. Clinical studies need to be done to evaluate these changes in growing children.

## Background

Rapamycin is a powerful immunosuppressant widely used in children to maintain the renal allograft. Studies have shown that rapamycin decreases cell proliferation by inhibition of the mammalian target of rapamycin (*mTOR*), a key regulator in cell growth. In addition, rapamycin has been demonstrated to exert anti-angiogenic properties to control tumor growth by reduction in vascular endothelial growth factor (*VEGF*) expression [1,2]. Due to its anti-proliferative effects, long-term rapamycin therapy may have adverse effects on linear growth in young children.

Investigators have reported that bone length decreased in young rats with normal renal function treated with rapamycin at 2 mg/kg daily for 14 days accompanied by alterations in growth plate architecture and lower chondrocyte proliferation assessed by bromodeoxyuridine (BrDU) incorporation [3,4]. Changes in trabecular bone modeling and remodeling with decrease in body length have been demonstrated in 10 week old rats after 2 weeks of rapamycin [3]. In contrast, Joffe and coworkers showed that a higher dose of rapamycin at 2.5 mg/kg per day for 14 days transiently lowered serum osteocalcin and calcitriol levels but it did not affect trabecular bone volume or bone formation rate [5]. Rapamycin inhibited osteoclast function, lessened bone resorption, decreased osteoblast proliferation and enhanced osteoblast differentiation in various in-vitro experiments [6-8].

Since rapamycin is now a standard immunosuppressant used to maintain an organ transplant in children, linear growth may be affected if rapamycin is administered long-term to young and growing patients. The aim of the current study is to assess the short and long-term effects of rapamycin on endochondral bone growth in young rats with normal renal function using markers of chondrocyte proliferation, chondrocyte differentiation, chondroclast/osteoclastic resorption and angiogenesis in the tibial growth plate.

## Methods

Twenty six male, 3 week old Sprague-Dawley rats with mean weight of 47 ± 4 grams, mean length of 20 ± 1 cm, were obtained from Harlan Laboratories (Indianapolis, IN), housed in individual cages at constant temperature with free access to drinking water. These are the approximate age comparisons between a rat and a child: a three week old weanling rat may be comparable to an infant and a rat between 5 to 7 weeks of age may approximate the age of a child. After 24 hours of acclimatization, the rats were randomly assigned to two groups: Rapamycin, N = 13, or Control, N = 13. Rapamycin was given at 2.5 mg/kg daily by gavage route and equal amount of saline was given to the Control group. The dose of rapamycin was based on previous published studies that demonstrated significant effects on body growth [4] and the length of treatment was adapted from our previous experiments that showed changes in the growth plate after 10 days of treatment [9]. Rapamycin and saline were given either for 2 weeks (Rapamycin-2 wks, N = 6; Control-2 wks, N = 6) or 4 weeks (Rapamycin-4 wks, N = 7; Control-4 wks, N = 7). All procedures were reviewed and approved by the Research Animal Resource Center at the University of Wisconsin and conducted in accordance with the accepted standards of humane animal care.

Rapamycin can lower oral intake which may subsequently affect growth [10]. To ensure equivalent caloric intake in all animals, the Rapamycin group was pair-fed to the Control animals by providing the amount of food each day to Control that had been consumed the previous day by the Rapamycin treated rats using a standard rodent diet (23.4% protein, 0.6% calcium, 0.6% phosphate) (Purina Mills, Indianapolis, IN).

Body weight was obtained weekly and body length was measured at the start and at the end of the 2 weeks or 4 weeks study period under sedation by measuring the distance from the tip of the nose to the end of the tail. At the end of the study period, the rats were anesthetized, killed by exsanguination and underwent trans-cardiac perfusion with 4% paraformaldehyde in phosphate buffered saline. Blood was obtained for determinations of serum calcium, creatinine, phosphate, urea nitrogen, parathyroid hormone (PTH) and insulin-like growth factor-I. Both tibiae from each animal were obtained and tibial length was measured between the proximal and distal articular surfaces using a caliper. Triplicate measurements were obtained for each bone, and the average of these determinations was taken to represent overall tibial length. Bones were decalcified in 15% ethylenediamine tetra-acetic acid in phosphate buffered saline, pH 7.4, at 4°C for approximately two weeks and embedded in paraffin. Five micrometer sections of bone were obtained for morphometric analysis, in-situ hybridization and immunohistochemistry studies.

### Serum biochemical determinations

Serum was obtained by centrifugation and samples were stored at -80°C until assays are done. Serum urea nitrogen, creatinine, calcium, and phosphate levels were measured using standard laboratory methods. Parathyroid hormone levels were measured using the Rat Bioactive Intact PTH ELISA assay kit (Immutopics, San Clemente, CA). IGF-I levels were measured using the Rat IGF-I ELISA assay kit (Diagnostic Systems Laboratories Inc, Webster, TX).

### Growth plate morphometry

The proximal growth plate of the tibia was selected for the experiments due to its fast growth [11]. For morphometric analysis, three 5-μm sections of bone were obtained from each tibia and stained with hematoxylin and eosin. Sections were viewed by light microscopy at 25× and images were captured onto a computer monitor. The total width of the growth plate cartilage at the proximal end of each tibia was measured at equally spaced intervals along an axis oriented 90° to the transverse plane of the growth plate and parallel to the longitudinal axis of the bone using an image-analysis software (Elektronik 200, Kontron Instruments, Ltd., Hallbergmoos, Germany) [9]. At least ten measurements were obtained from each epiphyseal growth plate. The width of the zones occupied by hypertrophic and proliferative chondrocytes was measured by the same method and the values are expressed as a ratio of the hypertrophic or proliferative zone to the total growth plate width.

### In-situ hybridization (radioactive)

For in-situ and immunohistochemistry experiments, individual sections of bone obtained from rats in each study group were mounted together on individual glass slides to permit valid side-by-side comparisons among samples from each group and to minimize differences that could be attributed to slide-to-slide variation during the specimen processing and development. Approximately 70–80 slides are included in each experiment.

In-situ hybridization was performed using methods described elsewhere [9]. Briefly, ^35^S-labeled sense and antisense riboprobes were generated encoding mouse *MMP-9/gelatinase B *(provided by Drs. G.V. Segre and K. Lee, MGH, Boston, MA) and rat *vascular endothelial growth factor *(provided by Dr. Zena Werb, University of California, San Francisco, CA) and labeled to a specific activity of 1–2 × 10^9 ^cpm/μg using the Gemini transcription kit (Promega Corp, Madison, WI). After hybridization and post-hybridization washing, the slides were exposed to x-ray film (Kodak Scientific Imaging Systems, Rochester, New York) overnight, and emulsion autoradiography was done using NTB-2 (Eastman Kodak, Rochester, New York) at 4°C.

Slides were viewed at 100× under bright field microscopy and the number of silver grains overlying each chondrocyte profile was counted using an image analysis system (Kontron Instruments, Hallbergmoos, Germany) [9]. In each specimen, fifty to sixty cell profiles (chondrocytes) were assessed in the layer of chondrocytes where mRNA was expressed and the results represent the average of these measurements. Data are expressed as the number of silver grains/1000 μm^2 ^of cell profile. To quantify *gelatinase B/MMP-9 *expression, the slides were viewed at 65× and the area with the silver grains was measured and expressed as percentage of the total area in the chondro-osseous junction.

### Immunohistochemistry experiments

Immunohistochemistry experiments were performed using methods described previously [12]. All primary antibodies were obtained from Santa Cruz Biotechnology unless indicated. Sections were deparaffinized, rehydrated, and immersed in 3% H_2_O_2 _and antigen was unmasked using either heat-induced epitope retrieval (10 mmol/L sodium citrate, pH 6.0 at 90°C for 5 min) or microwave for 5 minutes. Blocking was done using 5% goat serum at room temperature. After blocking, the appropriate primary antibody was added and incubated in 4°C overnight. The slides were washed in PBS, incubated with the goat anti-mouse biotin conjugate (Sigma Co, St. Louis, MO), then with extravidin-peroxidase (Sigma) and counterstained with either hematoxylin or 1% methylgreen.

The following primary antibodies were selected to evaluate chondrocyte proliferation: [a]*histone-4 *at 5 μg/ml, [b] *mammalian target of rapamycin (mTOR) at *4 μg/ml, [c] *parathyroid hormone/parathyroid hormone related-peptide *(*PTH/PTHrP*) at 4.4 μg/ml (Upstate Biotechnology), [d] *Growth Hormone Receptor (GHR) *at 4 μg/ml, and [e] *type II collagen (Col2a) *at 4 μg/ml. Chondrocyte maturation was assessed using: [a] *Indian Hedgehog (Ihh) *at 10 μg/ml, [b] *Insulin-like Growth Factor I (IGF-I) *at 10 μg/ml (Upstate Biotechnology], [c] *Insulin Growth Factor Binding Protein (IGFBP3) *at 10 μg/ml, [d] *p57*^*Kip*2 ^at 4 μg/ml, [e] *p21*^*Waf*1/*Cip*1 ^at 8 μg/ml, [f] *type *× *collagen (Col10a) *at 8 μg/ml, and [g] *Bone Morphogenetic Protein-7 *(*BMP-7*) at 5 μg/ml.

Osteo/chondroclastic activity was evaluated using *Receptor Activator for Nuclear Factor Kappa β Ligand (RANKL) *at 6 μg/ml and *Osteoprotegerin (OPG) *at 5 μg/ml. Histochemical staining for tartrate-resistant acid phosphatase (TRAP) and *gelatinase B/MMP-9 *were done using methods reported previously [9].

For quantification of the protein expression, slides were viewed at 65× by bright field microscopy and images were captured using a CCD video camera control unit. Approximately 50 to 60 cell profiles (chondrocytes) were assessed in the layer of the growth plate where the protein expression was counted and expressed as percentage of the labeled cells over the total number of cells where the expression is localized and the number of positive cells was counted and expressed as percentage of the labeled cells over the total number of cells where the expression is localized (Labeling Index) [12].

Histochemical staining for tartrate-resistant acid phosphatase (TRAP) was done using methods previously reported on sections of bone prepared and mounted in the same manner as for in-situ hybridization and immunohistochemistry experiments [9]. To quantify tartrate resistant acid phosphatase (TRAP), the number of TRAP positive cells in the chondro-osseous junction was counted and expressed as number of cells per area measured in the chondro-osseous junction and in the nearby primary spongiosa [9].

### Statistical analysis

All results are expressed as mean values ± 1 SD. Data were evaluated by one-way ANOVA and comparisons among groups were done using Bonferroni/DUNN post-hoc tests using the StatView^® ^statistical software (SAS Institute, Cary, NC). The Pearson product moment correlation coefficient was used to evaluate the relationship between two numerical variables. For all statistical tests, probability values less than 5% were considered to be significant.

## Results

### Measurements of body weight, body length and food intake

Gain in body weight was 14 percent and 19 percent higher in Control compared to Rapamycin groups after 2 and 4 weeks of treatment (Table 1). Body length measurements declined by 11 percent and 19 percent after 2 and 4 weeks of Rapamycin (Table 1). Tibial length measurements were 6 to 10 percent shorter in both Rapamycin groups (Table 1). Although the total caloric intake was similar in Rapamycin and Control groups, the calculated food efficiency ratio was higher with rapamycin which may suggest that a higher caloric intake may be required for growth or there may be dysregulation in the utilization of calories during rapamycin administration (Table 1).

**Table 1:** Morphometric measurements, food intake and food efficiency ratio (FER) in all groups

Parameters	2 weeks		4 weeks	
	
	Rapamycin, N = 6	Control, N = 6	Rapamycin, N = 7	Control, N = 7
Change in body weight^a ^(grams)	63 ± 6^d, e^	73 ± 8^d^	128 ± 7^f^	159 ± 11

Change in body length^a ^(centimeters)	7.6 ± 0.6^d, e^	8.6 ± 0.5^d^	13 ± 1.2^f^	16 ± 0.7

Tibial Length^b ^(centimeters)	3.0 ± 0.05^d, e^	3.2 ± 0.05^d^	3.4 ± 0.09^f^	3.7 ± 0.15

Total food intake^b ^(grams)	185 ± 1.8	183 ± 5.3	390 ± 32	410 ± 4.8

Food Efficiency Ratio^c ^(FER)	3.0 ± 0.5^e^	2.6 ± 0.6	3.0 ± 0.5^f^	2.6 ± 0.3

^a^Difference between measurements at 2 or 4 weeks and baseline

^b^Obtained at the time of sacrifice

^c^Total food consumed/weight gain during the study period

^d^p < 0.0001, 2 weeks versus 4 weeks (same group)

^e^p < 0.001, rapamycin versus control at 2 weeks

^f^p < 0.0001, rapamycin versus control at 4 weeks

### Serum biochemical parameters

Serum parathyroid hormone (PTH) and phosphate levels declined after 4 weeks of rapamycin (Table 2). Serum calcium levels were similar in all groups (Table 2). Serum creatinine levels were comparable in Rapamycin and Control groups at the end of 2 weeks and 4 weeks of treatment (Table 2). Serum IGF-I levels were 18 percent lower in Rapamycin and Control at the end of 2 weeks (Table 2).

**Table 2:** Biochemical parameters in all groups

Serum Parameters	2 weeks		4 weeks	
	
	Rapamycin, N = 6	Control, N = 6	Rapamycin, N = 7	Control, N = 7
Intact PTH^a ^(pg/mL)	40 ± 22	68 ± 34	32 ± 21^c^	90 ± 37

Calcium^a ^(mg/dL)	10 ± 0.6	10 ± 0.4	9.8 ± 0.1	10 ± 0.1

Phosphorus^a ^(mg/dL)	12 ± 0.2^b^	12 ± 0.5	10 ± 0.5	11 ± 0.7

Creatinine^a ^(mg/dL)	0.2 ± 0.04^b^	0.2 ± 0.03^b^	0.3 ± 0.04	0.3 ± 0.07

IGF-I^a ^(ng/mL)	708 ± 222^b^	867 ± 180^b^	1028 ± 256^c^	1318 ± 297

^a^Obtained at the time of sacrifice

^b^p < 0.003, 2 weeks versus 4 weeks (same group)

^c^p < 0.006, rapamycin versus control (4 weeks)

### Growth plate measurements

**Figure 1:**
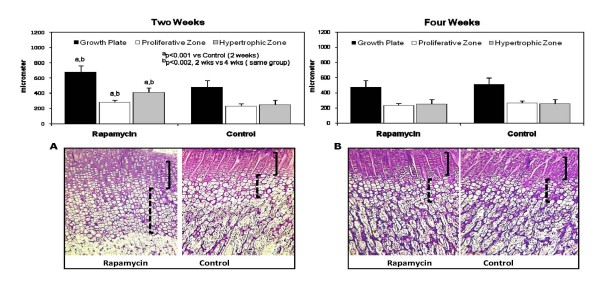
**The upper panels show the measurements of the total growth plate (black square), proliferative zone (white square) and hypertrophic zone (gray square) after 2 weeks (left panel) and after 4 weeks (right panel) in Rapamycin and Control groups, expressed in micrometers**. The lower panels show the corresponding photomicrographs of the growth plate cartilage in all groups, 30×. Note the increase in the zone occupied by hypertrophic chondrocytes in the 2 weeks Rapamycin group (left panel). The solid brackets denote the layer of proliferating chondrocytes and the dashed brackets indicate the hypertrophic zone; ^a^p < 0.001 Rapamycin (2 weeks) vs Control (2 weeks); ^b^p < 0.002 Rapamycin (2 weeks) vs Rapamycin (4 weeks).

Despite shorter body and tibial length, the growth plate was 26 percent wider compared to Control after two weeks of rapamycin accompanied by an increase in the area occupied by hypertrophic chondrocytes and a decrease in the proliferative zone (Figure 1). At the end of 4 weeks, the growth plate width was similar between the Rapamycin and the Control, 475 ± 89 μm and 509 ± 35 μm, p = NS (Figure 1). There were no obvious abnormalities in the columnar architecture of the growth plate cartilage (Figure 1).

### In-situ hybridization and immunohistochemistry studies

**Figure 2:**
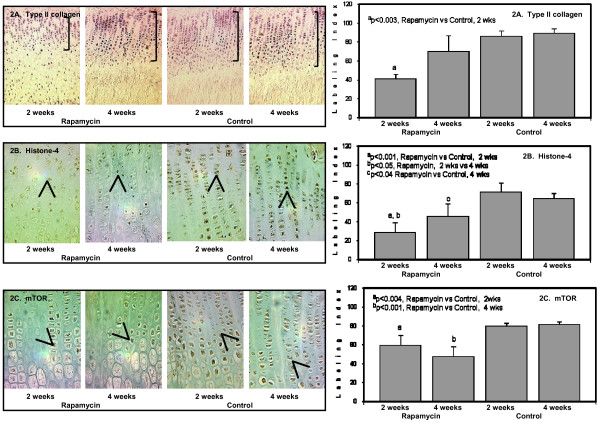
**The panels on the left show representative photomicrographs of *type II collagen *protein expression (2A) in the proliferating and hypertrophic chondrocytes, 20× (denoted by the purple color and brackets); ^a^p < 0.003 Rapamycin (2 weeks) vs Control (2 weeks)**. *Histone-4 *protein expression (2B, denoted by arrows and brown color), 50×; ^a^p < 0.001 Rapamycin (2 weeks) vs Control (2 weeks); ^b^p < 0.05 Rapamycin (2 weeks) vs Rapamycin (4 weeks); ^c^p < 0.04 Rapamycin (4 weeks) vs Control (4 weeks). *mTOR *protein expression (2C, denoted by arrows and brown color), 50×; ^a^p < 0.004 Rapamycin (2 weeks) vs Control (2 weeks); ^b^p < 0.001 Rapamycin (4 weeks) vs Control (4 weeks). The panels on the right show the quantification of the protein expression for *type II collagen *(upper panel); *histone-4 *(middle panel); and *mTOR *(lower panel) after 2 weeks and 4 weeks in Rapamycin and Control groups expressed as number of labeled cells to the total number of cells in the appropriate zone (Labeling Index).

Rapamycin inhibits the *mammalian target of rapamycin *(*mTOR*) which is crucial to cell cycle progression and thus, may lower chondrocyte proliferation. In the current study, we evaluated whether the shorter bone growth was primarily due to a decline in chondrocyte proliferation. The protein expression of selected markers associated with chondrocyte proliferation was assessed including *PTH/PTHrP receptor*, *histone-4*, *mTOR, growth hormone receptor *and *type II collagen *(*Col2a1*). In the growth plate, *Col2a1 *is the most abundant collagen which is expressed in all layers of chondrocytes. Rapamycin lowered *Col2a1 *expression by 40 percent compared to Control at 2 weeks particularly in the hypertrophic chondrocytes (Figure 2A). After 4 weeks of Rapamycin, *Col2a1 *staining was comparable to Control (Figure 2A).

*Histone-4 *localized to the proliferating chondrocytes and declined by 60 percent after 2 weeks of rapamycin compared to Control; 28 ± 11 percent versus 71 ± 10 percent (Figure 2B), p < 0.001. Similar to *Col2a1 *expression, *histone-4 *slightly increased after 4 weeks of rapamycin but remained 40 percent lower than Control (Figure 2B), p = < 0.05. Histone and DNA synthesis are initiated at the beginning of S phase of the cell cycle by cyclin/cdk2 activity. Cyclin expression was not evaluated in the current experiment, but our previous results have shown that *histone-4 *positively correlated with proliferating nuclear staining (PCNA) which is specific to proliferating cells [13].

*mTOR *expression was demonstrated in both proliferating and upper hypertrophic chondrocytes and declined after 2 and 4 weeks of rapamycin (Figure 2C).

**Figure 3:**
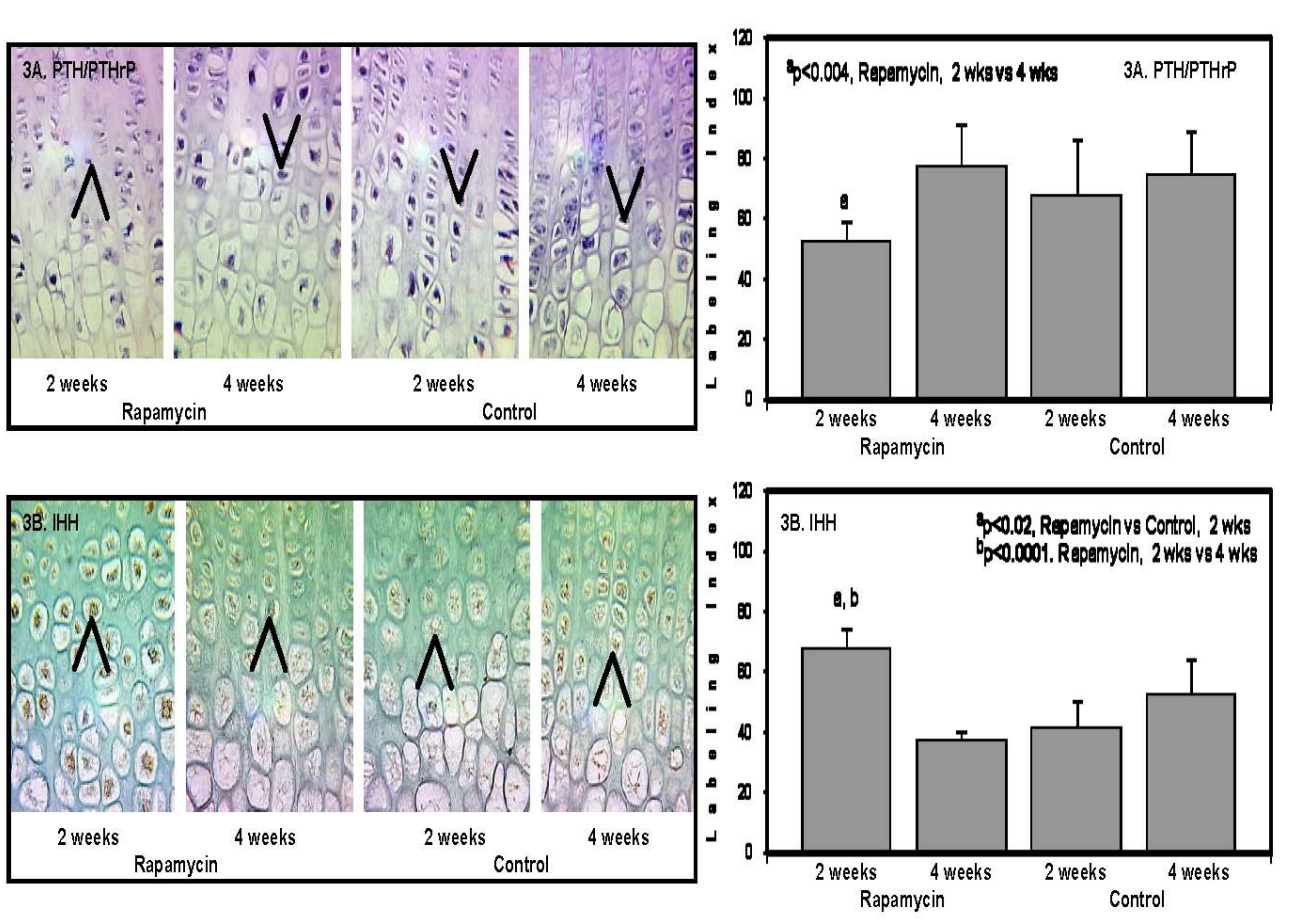
**The panels on the left show representative photomicrographs of *PTH/PTHrP *protein expression (3A) in the lower proliferating and upper hypertrophic chondrocytes, 50× (denoted by the purple color and arrows); ^a^p < 0.004 Rapamycin (2 weeks) vs Rapamycin (4 weeks)**. *Indian hedgehog *protein expression in the lower proliferating and upper hypertrophic chondrocytes (3B, denoted by arrows and brown color), 50×; ^a^p < 0.02 Rapamycin (2 weeks) vs Control (2 weeks); ^b^p < 0.0001 Rapamycin (2 weeks) vs Rapamycin (4 weeks). The panels on the right show the quantification of the protein expression for *PTH/PTHrP *(upper panel); and *Indian hedgehog *(lower panel) after 2 weeks and 4 weeks in Rapamycin and Control groups expressed as number of labeled cells to the total number of cells in the appropriate zone (Labeling Index).

*PTH/PTHrP *and *Ihh *are essential in the regulation of chondrocyte proliferation and chondrocyte differentiation in the growth plate cartilage. A feedback loop exists between *PTHrP *and *Ihh *which controls the pace of chondrocyte proliferation [14]. Acceleration of chondrocyte differentiation and premature ossification in the growth plate have been reported in *PTH/PTHrP *null mouse [15,16]. Chondrocyte proliferation declined and the area occupied by hypertrophic chondrocytes increased in targeted deletion of *Ihh *[14]. After 2 weeks of rapamycin, *PTH/PTHrP *which localized to the lower proliferating and upper hypertrophic chondrocytes declined by 30 percent compared to Control. In contrast, *Ihh *expression confined mostly to the hypertrophic chondrocytes increased approximately 2-fold after 2 weeks of rapamycin (Figure 3A). At the end of 4 weeks, *PTH/PTHrP *and *Ihh *expression were comparable to the Control group (Figure 3A and 3B). The current results suggest that the widening of the hypertrophic zone and decrease in the proliferative zone may be due in part to enhancement of *Ihh *and downregulation of *PTH/PTHrP*.

Other markers used in the study to assess chondrocyte maturation include: *IGF-I *protein, *IGF-I binding protein-3 *(*IGFBP3*), *type *× collagen and *bone morphogenetic-7 *(*BMP-7*).

**Figure 4:**
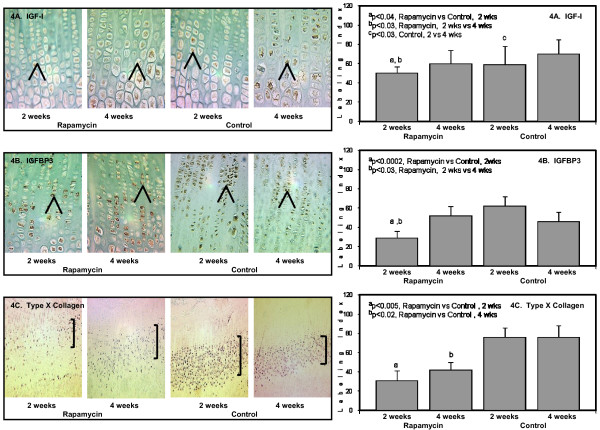
**The panels on the left show representative photomicrographs of *IGF-I *protein expression (4A), 50× (denoted by the brown color and arrows); ^a^p < 0.04 Rapamycin (2 weeks) vs Control (2 weeks); ^b^p < 0.03 Rapamycin (2 weeks) vs Rapamycin (4 weeks); ^c^p < 0.03 Control (2 weeks) vs Control (4 weeks)**. *IGFBP3 *protein expression (4B, denoted by arrows and brown color), 50×; ^a^p < 0.002 Rapamycin (2 weeks) vs Control (2 weeks); ^b^p < 0.03 Rapamycin (2 weeks) vs Rapamycin (4 weeks). *Type *× *collagen (col10a) *protein expression localized only to the hypertrophic chondrocytes (4C, denoted by purple color and brackets), 20×; ^a^p < 0.005 Rapamycin (2 weeks) vs Control (2 weeks); ^b^p < 0.02 Rapamycin (4 weeks) vs Control (4 weeks). The panels on the right show the quantification of the protein expression for *IGF-I *(upper panel); *IGFBP3 *(middle panel); and *type *× *collagen *(lower panel) after 2 weeks and 4 weeks in Rapamycin and Control groups expressed as number of labeled cells to the total number of cells in the appropriate zone (Labeling Index).

The protein expression of *IGF-I *which was restricted to the hypertrophic chondrocytes decreased after 2 weeks of rapamycin compared to Control (Figure 4A). In agreement with other published studies, *IGF-I *staining was 20 percent lower in the 2 weeks Control animals compared to 4 weeks Control (Figure 4A). *IGF-II *and not *IGF-I *has been demonstrated to be more abundant in younger animals and that *IGF-I *may be associated with chondrocyte hypertrophy and mineralization [17]. The expression of IGF-II was not assessed in the current study.

*IGFBP3 *protein expression was localized to the proliferating and upper hypertrophic chondrocytes in both 2 weeks and 4 weeks Rapamycin and Control groups (Figure 4B). Two weeks of rapamycin downregulated *IGFBP3 *by 53 percent compared to the Control group, and by 44 percent compared to the 4 weeks Rapamycin group (Figure 4b). The changes in *IGFBP3 *were similar to the changes in *IGF-I *protein expression.

*Type *× *collagen *(*col10a*) is a marker of chondrocyte maturation and solely localized to the hypertrophic chondrocytes. Although the width of the zone occupied by the hypertrophic chondrocytes increased with rapamycin, *col10a *expression declined 2-fold after 2 and 4 weeks of treatment compared to Control groups (Figure 4C).

**Figure 5:**
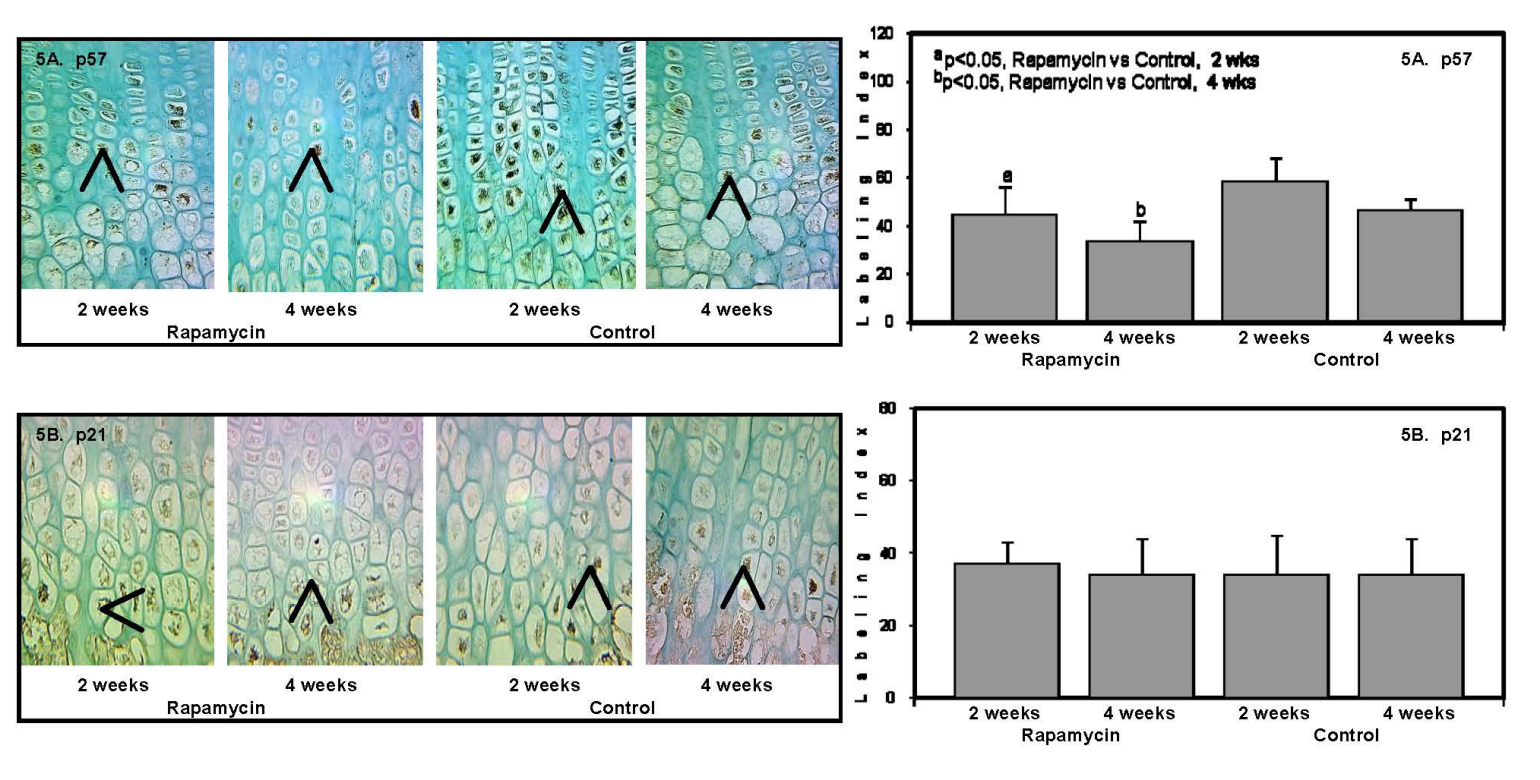
**The panels on the left show representative photomicrographs of *p57*^*Kip*2 ^protein expression in the hypertrophic chondrocytes (5A, denoted by arrows and brown color), 50×; ^a^p < 0.05 Rapamycin (2 weeks) vs Control (2 weeks); ^b^p < 0.05 Rapamycin (4 weeks) vs Control (4 weeks)**. *p21*^*Cip*-1/*SDI*-1/*WAF*-1 ^protein expression localized only to the terminal hypertrophic chondrocytes (5B, denoted by arrows and brown color), 50×. The panels on the right show the quantification of the protein expression for *p57*^*Kip*2 ^(upper panel); and *p21*^*Cip*-1/*SDI*-1/*WAF*-1 ^(lower panel) after 2 weeks and 4 weeks in Rapamycin and Control groups expressed as number of labeled cells to the total number of cells in the appropriate zone (Labeling Index).

It has been demonstrated that the proliferative actions of *PTHrP *may be mediated by downregulation of cyclin kinase inhibitors *p57*^*Kip*2 ^and *p27*^*Kip*1 ^[18]. In the current study, there was a 20 to 30 percent reduction in *p57*^*Kip*2 ^staining in the hypertrophic chondrocytes of both Rapamycin groups (2 and 4 weeks) compared to Control accompanied by lower *histone-4 *expression (Figure 5A). There were no changes in *p21*^*Cip*-1/*SDI*-1/*WAF*-1 ^expression in all groups (Figure 5B). The expression of *bone morphogenetic protein-7 *(*BMP-7*) and *growth hormone receptor *did not differ among groups (pictures not included).

**Figure 6:**
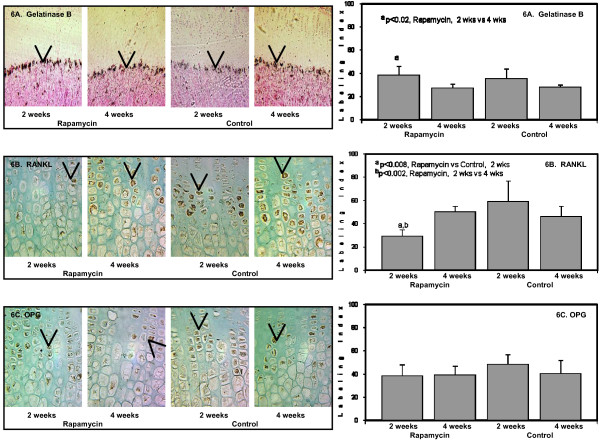
**The panels on the left show representative photomicrographs of *gelatinase B/MMP-9 *mRNA expression in the chondro-osseous junction (6A), denoted by the black color and arrows, 20×); ^a^p < 0.02 Rapamycin (2 weeks) vs Rapamycin (4 weeks)**. *RANKL *protein expression in the hypertrophic chondrocytes (6B, denoted by arrows and brown color), 50×; ^a^p < 0.008 Rapamycin (2 weeks) vs Control (2 weeks); ^b^p < 0.002 Rapamycin (2 weeks) vs Rapamycin (4 weeks). *OPG *protein expression localized to the hypertrophic chondrocytes (6C, denoted by arrows and brown color), 50×. The panels on the right show the quantification of the protein expression for *gelatinase B/MMP-9 *(upper panel); *RANKL *(middle panel); and *OPG *(lower panel) after 2 weeks and 4 weeks in Rapamycin and Control groups expressed as number of labeled cells to the total number of cells in the appropriate zone (Labeling Index).

Vascular invasion and cartilage resorption are crucial steps in endochondral bone growth. Rapamycin did not affect the expression of g*elatinase B *or *matrix metalloproteinase-9 *(*MMP-9*) mRNA after 2 or 4 weeks compared to the Control groups, although the expression was relatively higher in the growth plate of younger animals (Figure 6A).

*Receptor activator of nuclear factor-kappa β ligand *(*RANKL*) and *osteoprotegerin *(*OPG*) participate in the regulation of osteo/chondroclastogenesis. We have previously demonstrated that *RANKL *and *OPG *expression were localized to the hypertrophic chondrocytes and the ratio between RANKL:OPG has been used to estimate the presence of osteo/chondroclast differentiation [19]. There was a 40 percent decrease in *RANKL *expression after 2 weeks of rapamycin compared to Control (Figure 6B); this change was not evident after 4 weeks of rapamycin (Figure 6B). Since *OPG *expression did not change in all groups (Figure 6C), the RANKL:OPG ratio was lower in the 2 week rapamycin group which may suggest decline in osteo/chondroclastogenesis (Figure 6C).

**Figure 7:**
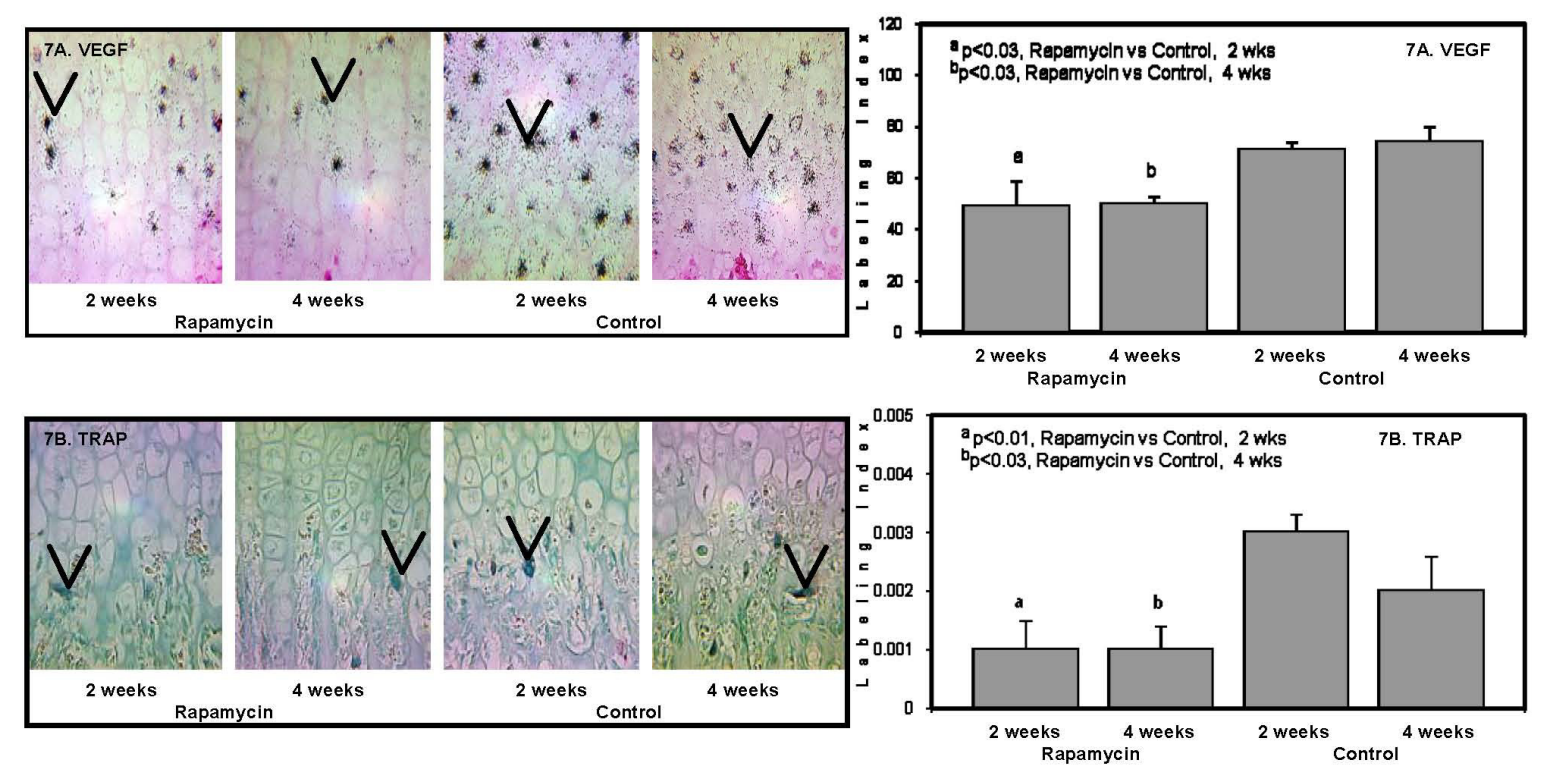
**The panels on the left show representative photomicrographs of *VEGF *mRNA expression in the hypertrophic chondrocytes (7A), denoted by the black color and arrows, 50×; ^a^p < 0.03 Rapamycin (2 weeks) vs Control (2 weeks); ^b^p < 0.03 Rapamycin (4 weeks) vs Control (4 weeks)**. TRAP positive chondro/osteoclasts (7B), denoted by arrows and red color, 50×; ^a^p < 0.01 Rapamycin (2 weeks) vs Control (2 weeks); ^b^p < 0.03 Rapamycin (4 weeks) vs Control (4 weeks). The panels on the right show the quantification of the protein expression for *VEGF *(upper panel) and *TRAP *(lower panel) after 2 weeks and 4 weeks in Rapamycin and Control groups expressed as number of labeled cells to the total number of cells in the appropriate zone (Labeling Index).

*Vascular endothelial growth factor *(*VEGF*) was demonstrated in the mature hypertrophic chondrocytes and the expression was 30 percent less after 2 and 4 weeks of rapamycin compared to control (Figure 7A). Histochemical staining for tartrate resistant acid phosphatase (TRAP) was considerably reduced in both rapamycin groups (Figure 7B).

## Discussion

Rapamycin is a potent immunosuppressant which can inhibit endochondral bone growth in young rats. Our study suggests that rapamycin may decrease chondrocyte proliferation, alter maturation of hypertrophic chondrocytes, delay vascular invasion and reduce TRAP activity in the chondro-osseous junction of the growth plate cartilage.

Currently, there are no available studies that have evaluated the effects of rapamycin in young and growing children. The implications of our findings on linear growth need further evaluation in young children who are maintained on long-term immunosuppressant treatment with rapamycin. The rapamycin dose used in the current study was higher than the currently prescribed amount in pediatric patients, but similar doses were previously utilized in published animal studies [3,4].

The adverse effects of rapamycin on the growth plate were more evident in younger animals. It was expected that the smaller animals which were treated with 2 weeks of rapamycin will have smaller growth plate cartilage however, our findings demonstrated an increase rather than decrease in the total growth plate with widening of the layer occupied by hypertrophic chondrocytes. Although there was a significant increase in hypertrophic zone, the columnar architecture was preserved. The enlargement of the hypertrophic zone may be due in part, to a reduction in the number of proliferating chondrocytes, lower cartilage resorption in the chondro-osseous junction due to a decline in TRAP and there may be a delay in vascular invasion. Although the changes in the growth plate which were evident after 2 weeks improved at the end of 4 weeks of rapamycin, body length and tibial length measurements remained short. Longer follow-up needs to be done in future studies to assess whether catch-up growth will occur in the rapamycin treated animals.

The immunosuppressive effects of rapamycin are based on its ability to inhibit cell cycle progression from G_1 _to S phase and hinder DNA synthesis by restraining the phosphorylation of p70^S6 ^kinase leading to inactivation of the mammalian target of rapamycin (*mTOR*) [20,21]. The *mammalian target of rapamycin *(*mTOR*) integrates signals from nutrition and growth factors to coordinate cell growth and cell proliferation [22]. Rapamycin can also decrease cyclin D and cyclin E protein expression including downstream effectors involved in cell cycle progression [23]. In the present study, chondrocyte proliferation assessed by *histone-4 *and *mTOR *expression was significantly decreased. Although the markers of chondrocyte proliferation improved in older rats treated with rapamycin, bone length remained short after 7 weeks of study period. These findings suggest that the inhibitory effects of rapamycin on chondrocyte proliferation may be more significant in young animals due to rapid growth which may be a concern during long-term rapamycin therapy in young pediatric patients. The reduction in *histone-4 *and *mTOR *was also accompanied by a decline in *type II *(*col2a*) collagen expression, another marker of chondrocyte proliferation and important in the extracellular matrix support of chondrocytes.

The present study showed a downregulation of *PTH/PTHrP *accompanied by enhancement of *Ihh *after 2 weeks of rapamycin; such changes were not significant at the end of 4 weeks. The *PTH/PTHrP *and *Indian hedgehog *(*Ihh*) feedback loop plays an important role in chondrocyte proliferation and differentiation. The increase in the zone occupied by the hypertrophic chondrocytes may be a combination of the decline in *PTH/PTHrP *and upregulation of *Ihh *expression. Our current findings show that the downregulation of *PTH/PTHrP *during rapamycin therapy was not due to the enhancement of cyclin kinase inhibitor p*57*^*Kip*2^.

Chondrocyte proliferation, chondrocyte maturation and apoptosis of the terminal hypertrophic chondrocytes must be precisely coordinated and any delay in each stage can lead to shorter bone growth as shown in the current experiment. Markers of chondrocyte differentiation that were evaluated in the current paper including *IGF-I *and *IGF binding protein-3 *were downregulated after 2 weeks but improved at the end of 4 weeks. Only *type *× *collagen *(*col10*) and *p57*^*Kip*2 ^expression remained low after 4 weeks of rapamycin treatment. *Type *× *collagen *has been demonstrated to play an essential role in the initiation of matrix mineralization in the chondro-osseous junction and in the maintenance of progenitor cells for osteo/chondrogenesis and hematopoiesis [24]. The alterations in proliferation and differentiation of chondrocytes in the growth plate during rapamycin therapy may delay mineralization and vascularization in the appendicular skeleton and consequently, may affect the production of bone marrow progenitor cells. These findings will require further evaluation.

Alvarez and colleagues have demonstrated that 14 days of intraperitoneal rapamycin led to smaller tibial bones associated with decreased body weight and lower food efficiency ratio [4]. Our findings agree with previous reports and may suggest that during rapamycin treatment, animals may require higher amount of calories per day in order to grow. Since *mTOR *is an important modulator of insulin-mediated glucose metabolism, rapamycin may exert adverse effects on the absorption of nutrients [25]. When given orally as in the current study, rapamycin may lower intestinal absorption of glucose, amino acids and linoleic acids by decreasing the area of the absorptive intestinal mucosa [10].

Rapamycin has been studied as an effective treatment for cancer not only due to its anti-proliferative actions but for its anti-angiogenic properties [26]. Our current findings showed a significant downregulation of *vascular endothelial growth factor *expression in the hypertrophic chondrocytes of animals treated with rapamycin. Our findings are in agreement with previous reports by Alvarez-Garcia and coworkers [4]. Although there were no changes in *gelatinase B/MMP-9 *mRNA expression in the chondro-osseous junction, there was a considerable reduction in the number of TRAP positive chondro/osteoclasts suggesting that cartilage resorption may be altered by rapamycin. The delay in cartilage resorption and changes in chondro/osteoclast function may be due to the reduction in *RANKL *expression as shown in the present experiment and by other investigators [6]. There were no changes in *osteoprotegerin *(*OPG*) staining so *RANKL/OPG *ratio was lower compared to Control. The decrease in *RANKL/OPG *ratio may reflect a decrease in chondro/osteoclast recruitment and differentiation.

## Conclusion

Rapamycin is a novel and powerful immunosuppressant widely used in pediatric renal transplant recipients to maintain the allograft. We have shown in the current study that rapamycin can inhibit endochondral bone growth in a rapidly growing young animal. The shorter bone growth may be due in part, to the decline in chondrocyte proliferation, enhancement of chondrocyte maturation, and alterations in cartilage resorption and vascularization. Our findings have also demonstrated that the 2 week effects of rapamycin on chondrocyte proliferation, chondrocyte maturation and vascular invasion may improve to near normal if rapamycin is administered continuously as the animal matures although, no catch-up growth was demonstrated. The results in the current study may be limited by the semi-quantitative results obtained using in-situ and immunohistochemistry methods, so future experiments should be done using quantitative proteomic and genomic techniques. In addition, clinical studies are needed to assess whether long-term therapy with rapamycin can affect linear growth in young pediatric patients.

## Competing interests

The authors declare that they have no competing interests.

## Authors' contributions

CPS conceived the experimental design, drafted the manuscript, analyzed the results and performed the histological and morphometric measurements of the growth plate. YHZ participated in the study design, interpretation of the results and performed most of the in-situ and immunohistochemistry experiments. The authors read and approved the final manuscript.

## Pre-publication history

The pre-publication history for this paper can be accessed here:


